# Surface-Induced Photophysical Enhancement of Conjugated Polyelectrolytes on Cellulose Nanocrystals: A Single-Particle Study

**DOI:** 10.1021/acs.langmuir.5c03918

**Published:** 2025-10-10

**Authors:** Nour Merhi, Pierre Karam

**Affiliations:** Department of Chemistry, 11238 American University of Beirut, P.O. Box 11-0236, Beirut 1107 2020, Lebanon

## Abstract

Conjugated polyelectrolytes possess unique photophysical properties, yet their brightness and photostability are significantly hindered in water as they adopt a collapsed state. In this study, we present a strategy to overcome these limitations by assembling short poly­(phenylene ethynylene)-based conjugated polyelectrolytes onto cellulose nanocrystals, forming brighter fluorescent hybrid nanoparticles. This assembly process suppresses excimer-like species and promotes disaggregated, highly fluorescent, and photostable conformation whose emission is shifted to 450 nm. By analyzing the intensity-time traces and emission spectra at both the ensemble and single-particle levels, we unraveled the underlying mechanism of the enhanced photophysical properties of the conjugated polyelectrolytes. The cellulose nanocrystal surface facilitated the assembly of individual polymer chains into ordered aggregates, leading to improved overall photophysical properties. These findings highlight the potential of cellulose-based nanostructures as a platform for tuning and stabilizing the emission properties of conjugated materials in aqueous media.

## Graphical abstract

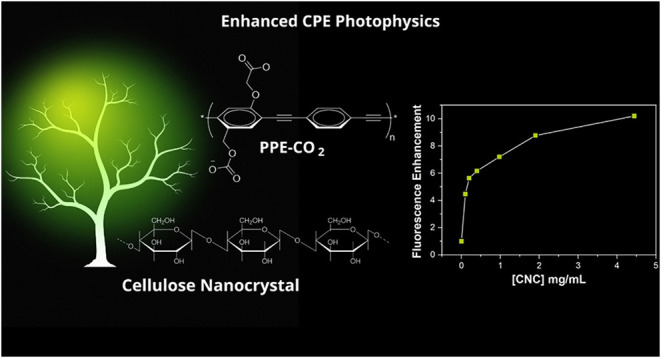

## Introduction

The development of new materials for applications in optoelectronics, biomedical imaging, and engineering has sparked significant interest in developing and improving the photophysical properties of fluorescent dyes, specifically conjugated polyelectrolytes (CPEs).^1–3^ This class of fluorescent polymers is known for its unique electronic, optical, and photophysical properties,^4,5^ making them valuable for diverse applications from biosensors to optoelectronic devices.^4,6–9^ Our laboratory has exploited these unique properties to develop temperature-sensitive nanoprobes and hybrid particles with exceptional photostability.^10^ Others formulated the CPEs into antibacterial material, biosensing, and bioimaging probes.^11–13^ However, despite this wide range of applications, CPEs still exhibit unfavorable photophysical properties when placed in polar solvents. Their hydrophobic backbone promotes aggregation. It often leads to collapsed conformations, which result in excimer-like emission. These species are characterized by broad, featureless, and red-shifted emission spectra with low quantum yield and accelerated photodegradation.^14^ To mitigate these effects, many attempts have been reported to reduce the aggregation-induced excimer formation by introducing chemical modifications to their water-soluble side groups,^15^ or by preparing them in a matrix locked in an optimal configuration.^16^

Herein, we demonstrate how cellulose nanocrystals can enhance the photophysical properties of short poly­(phenylene ethynylene)-based conjugated polyelectrolytes modified with carboxylic acid as side chains (PPE-CO_2_). While widely used, this class of conjugated polyelectrolytes is still hindered by conformation-induced photophysical limitations in aqueous environments. In water, PPE-CO_2_ tends to aggregate, leading to an excimer-like emission with a maximum of 520 nm. However, when prepared in an organic polar solvent, the emission shifts to 430 nm, accompanied by an improvement in the fluorescence emission. Given their ability to modulate molecular interactions, cellulose nanocrystals’ surface offers a promising approach to improving the photophysical properties of the poly­(phenylene ethynylene)-based conjugated polyelectrolyte. These biodegradable nanoparticles possess exceptional mechanical and chemical properties.^17–20^ Derived from trees and plants, they offer a sustainable alternative to synthetic polymers, making them a great choice for developing eco-friendly materials.^21,22^ Their high surface area and tunable chemistry make them particularly effective as scaffolds for immobilizing fluorescent dyes.^23–26^ By incorporating fluorescent molecules into cellulose nanocrystal-based composites, new materials were developed with enhanced optical properties.^27–31^ For instance, cellulose nanocrystals have been used to disaggregate, especially hydrophobic fluorophores, thus reducing the aggregation-induced fluorescence quenching. By covalently attaching carbon dots to cellulose nanocrystal surfaces, for example, highly emissive fluorescent films and hydrogels were prepared.^32^ This modification resulted in a large reduction in the aggregation of the fluorescent dots. Thioflavin T’s emission was also dramatically enhanced when mixed with cellulose.^33^ The improved photophysical properties were also instrumental in the detection of amyloid-like structures.

In this work, we tuned the photophysical properties of polyphenylene-based CPEs by assembling them onto the cellulose nanocrystals’ surface. The interaction shifted the fluorescence emission of PPE-CO_2_ to a more emissive species at 450 nm and significantly improved its photostability. Single-particle studies provided molecular-level insights into the conformation of the conjugated polyelectrolyte. By monitoring the changes in the fluorescence intensity and the emission spectra over time of single CNC/PPE-CO_2_ hybrid nanoparticles, we were able to confirm that the PPE-CO_2_ is assembled onto the cellulose nanocrystals’ surface as ordered chains that do not emit from excimer-like species. The prepared particles can prove instrumental in sensing and imaging applications.

## Results and Discussion

### CNC/PPE-CO_2_ Interactions at the Ensemble Level

Ensemble and single-particle spectroscopic methods were used to investigate the impact of cellulose nanocrystals on PPE-CO_2_ conjugated polyelectrolytes. We chose PPE-CO_2_ with an average of 108 repeating units, as it consistently exhibited stronger interactions when mixed with other macromolecules compared to shorter conjugated polyelectrolytes previously reported by our laboratory.^34–37^

The interaction between the conjugated polyelectrolytes and the cellulose nanocrystal was initially studied using dynamic light scattering (DLS) and electrophoretic mobility. Upon addition of PPE-CO_2_ to the CNC nanoparticle suspension, the zeta potential (*z*) shifted from −16.69 to −27.66 mV. This change is consistent with the increase in negative surface charge density associated with the adsorption of PPE-CO_2_ chains. The apparent hydrodynamic diameter decreased from 196 to 186 nm. Given the inherent rod-like geometry of CNCs, this small shift likely reflects reduced secondary agglomeration and/or compression of the hydration layer following the CPE binding. Together, the DLS and the zeta potential results confirm the assembly of the PPE-CO_2_ chains onto the nanocellulose.

Absorbance spectra were recorded before and after the addition of cellulose nanocrystals, as shown in Figure 1A. Upon introducing the nanocrystals, a redshift and a narrower peak were observed, indicating an increase in conjugation length and a more planar molecular conformation. Similar spectral shifts have been previously reported for poly­(phenylene ethynylene)-based conjugated polyelectrolytes, where changes in the absorption were attributed to variations in chain conformation, polymer aggregation, and interactions with amphiphilic or charged macromolecules.^38–42^ For instance, Silva et al. reported a significant improvement in the molecular alignment and planarity of poly­(3-hexylthiophene) (P3HT) in the presence of CNCs,^43^ leading to reduced torsional disorder and narrower bandwidth energy values associated with π–π* electronic transitions. These structural changes also resulted in a red shift in the absorption spectra and enhanced charge transport and conductivity in the conjugated polymer.

**Figure 1:**
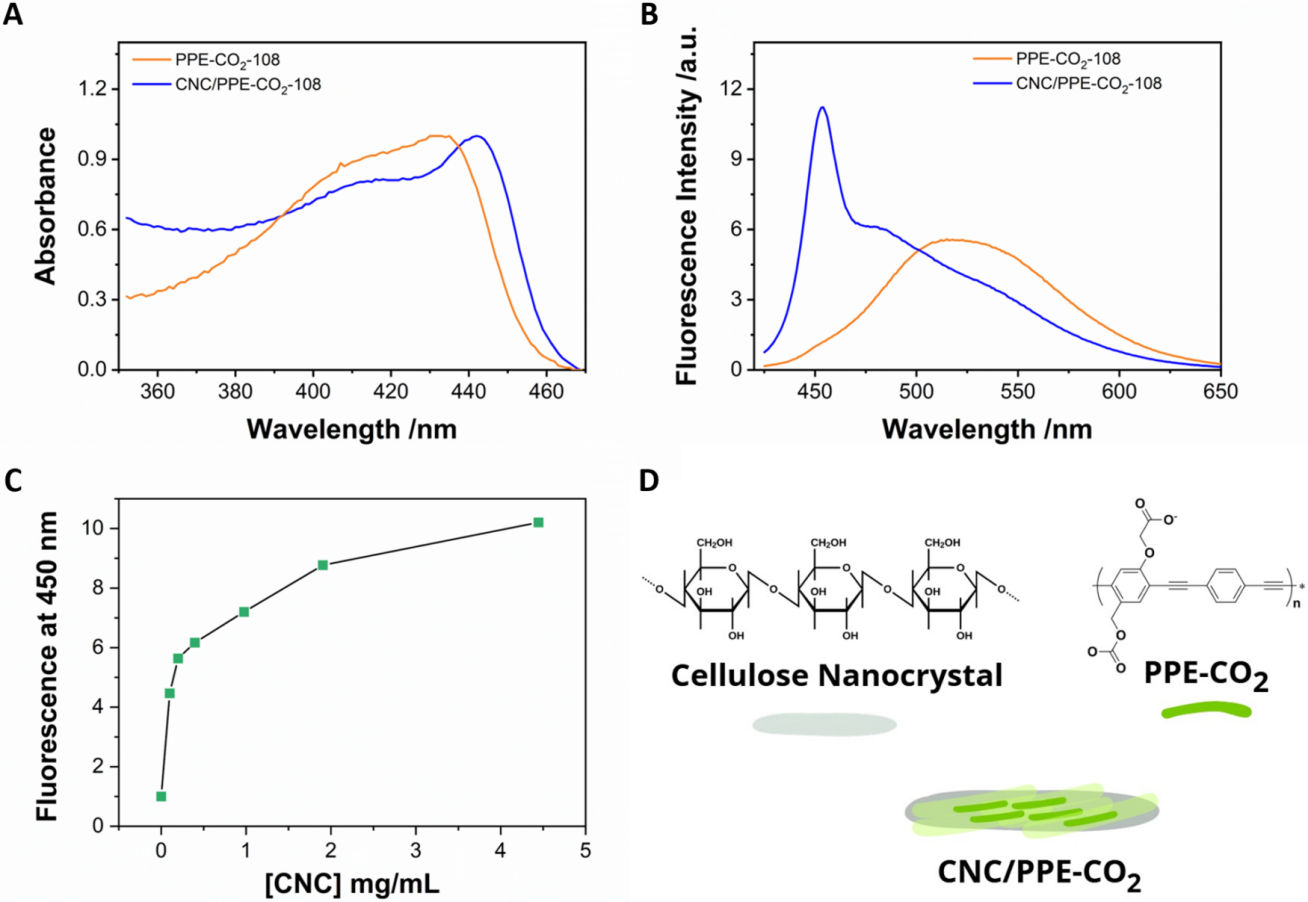
(A) Absorption spectra of PPE-CO_2_-108 in HEPES with NaCl before (orange) and after (blue) the addition of 4.5 mg/mL cellulose nanocrystals. (B) Fluorescence emission spectra of PPE-CO_2_-108 before (orange) and after (blue) the addition of 4.5 mg/mL cellulose nanocrystals. (C) Fluorescence changes recorded at 450 nm upon increasing cellulose nanocrystal concentration. Lines are used for visual aid. All experiments were done in 10 mM HEPES (pH = 7.0) with 150 mM NaCl. (D) Schematic and chemical representation of cellulose nanocrystal (CNC) and poly­(phenylene ethynylene) conjugated polyelectrolytes with carboxylic acid side groups (PPE-CO_2_).

Next, the fluorescence emission spectra were recorded. As stated earlier, the emission of PPE-CO_2_ is highly sensitive to its conformation and solvation state. In the absence of cellulose nanocrystals, the fluorescence emission spectra of PPE-CO_2_-108 exhibited a structureless peak at 520 nm. This spectral characteristic suggests the formation of excimer-like species through polymer aggregation. Upon introducing cellulose nanocrystals, a new emission peak is observed at 450 nm, concomitant with a significant increase in fluorescence intensity (Figures 1B,C and S1). This change reflects a transition to a more emissive conformation rather than a uniform enhancement across the full emission spectra. We selected 450 nm as the monitoring wavelength in Figure 1C to track the change in emission since this wavelength specifically tracks the formation of the desired, highly emissive conformation while excluding contributions from the excimer-like species at 520 nm. It is important to note here that both the absorbance and emission of CNC/PPE-CO_2_-108 (Max Abs.: 442 nm and max emission: 453 nm) differ from those of well-dissolved and individual chains of PPE-CO_2_ in methanol (Max Abs.: 411 nm and max emission: 432 nm; Figure S2). The maximum absorbance and emission of the hybrid nanoparticles are red-shifted compared to the single-chain solution. These measurements suggest that the cellulose surface does not completely disaggregate the conjugated polyelectrolyte into individual chains. Instead, it appears to promote a polymer configuration with emission characteristic of a more ordered structural state.^44,45^ In fact, the cellulose nanocrystals’ surface has a high density of hydroxyl groups, which can readily form hydrogen bonds with the carboxylic acid side chains of PPE-CO_2_, leading to their desegregation and stabilization.^46^ Additionally, the partial hydrophobicity of the cellulose backbone may interact and further stabilize the hydrophobic backbone of the conjugated polyelectrolyte. While our data support this interaction as the basis for suppressed excimer formation, a detailed atomistic picture will be the subject of future studies.

Conjugated polyelectrolytes suffer from low photostability, which can limit their applications. When free in solution, PPE-CO_2_-108 solution loses approximately 14% (at 520 nm) of its initial fluorescence intensity after 15 min of continuous irradiation. The full spectrum recorded before and after illumination (Figure S4) also confirms the photodegradation. In contrast, under the same experimental conditions, the CNC/PPE-CO_2_-108 hybrid particles exhibited an 80% increase in fluorescence intensity at 450 nm (Figure 2A). Specifically, the emission maximum remained centered at 450 nm, and no excimer-like emission around 520 nm was detected. This rules out detachment and reaggregation of the CPE chains of the CNC particles. In fact, the emission of CNC/PPE-CO_2_-108 before and after indicates a decrease in intensity in the red region, which could be reminiscent of some excimer species (Figure S5). We and others have reported similar observations, for instance, when MPS-PPV was complexed with PVP.^47,48^ We proposed that conjugated polyelectrolyte chains behave as multiple connected chromophores, resembling highly concentrated fluorophore solutions.^47,48^ Efficient energy transfer between chromophores to lower-energy or nonemissive trap sites quenches the overall intensity, and continuous irradiation progressively deactivates these sites, which can potentially explain the observed fluorescence enhancement. Moreover, growing evidence suggests that CPEs undergo chain scission under irradiation,^49^ which could further contribute to the observed increase in fluorescence intensity. This may also be a contributing mechanism since a reduction of CPE scission is expected when the polymers are assembled onto nanoparticles compared to free CPEs interacting in solution. While this provides a plausible explanation, we acknowledge that other processes, such as stabilization of emissive conformations on the CNC surface, may also contribute.

**Figure 2:**
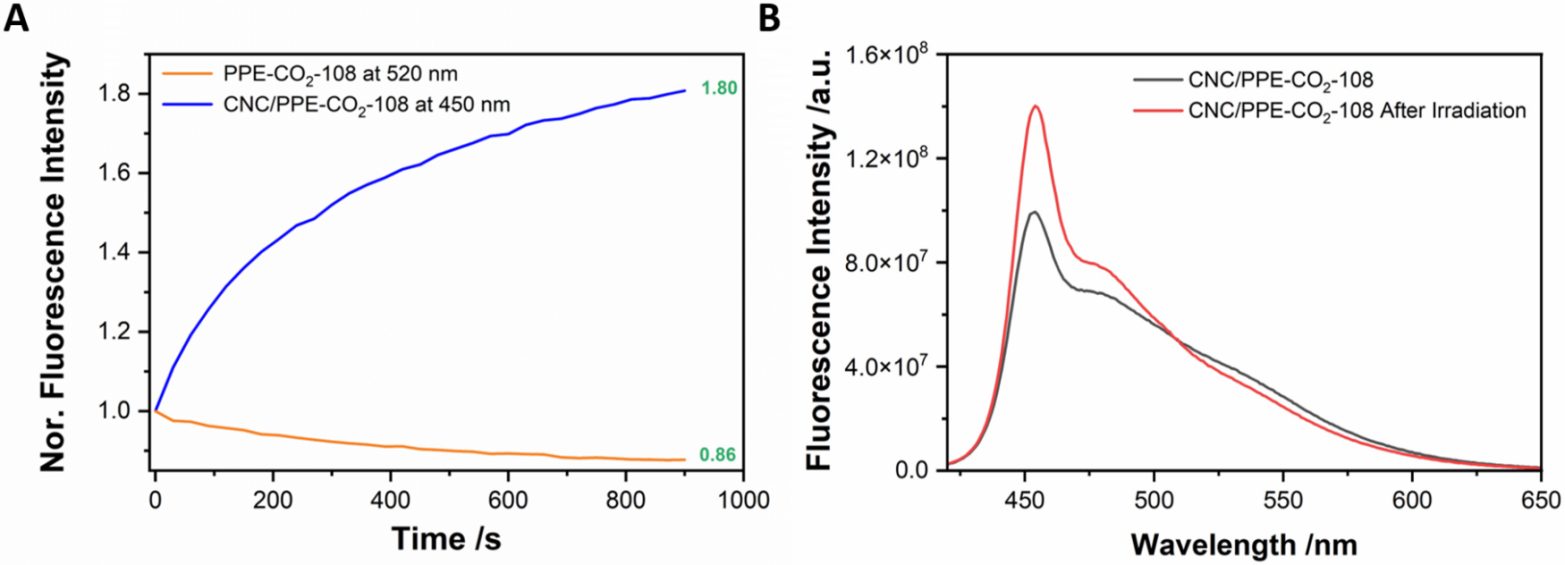
(A) Fluorescence intensity changes over time upon continuous irradiation of PPE-CO_2_-108 and CNC/PPE-CO_2_-108 in solution. (B) The emission spectra of CNC/PPE-CO_2_-108 before and after continuous irradiation.

Previous studies have demonstrated that cellulose nanocrystals can improve the properties of conjugated polymers through their surface-induced interactions. Alam et al. reported enhanced photoluminescence and extended lifetimes in P3HT embedded within a poly­(butyl acrylate) (PBA)-grafted cellulose nanocrystal matrix.^50^ Chang et al. grafted polyfluorene (PF) directly onto CNC surfaces, observing solid-state-like fluorescence properties despite having the PF-hybrid dispersed in solution. This effect was attributed to the restricted polymer chain mobility imposed by the nanoparticles, which enhanced local interactions of neighboring chromophores.^51^ Furthermore, Alam et al. functionalized CNCs with octacarboxylated cobalt phthalocyanine (CoPc),^51,52^ creating a hybrid material with exceptional photocatalytic activity. The CNC-CoPc complex exhibited enhanced charge separation, reduced aggregation, increased visible-light absorption, and suppressed radiative recombination, properties uncommon for typical semiconducting-insulator systems.

The fluorescence ensemble experiments of CNC/PPE-CO_2_-108 revealed improved photophysical properties of the conjugated polyelectrolytes when complexed onto the cellulose nanocrystals’ structures. However, ensemble experiments are limited to the average value, especially when an inherently heterogeneous solution like a conjugated polyelectrolyte is being studied. To gain better insights into the underlying mechanisms of the improved photophysical properties of these complexes, we performed single-particle spectroscopy, allowing us to probe the fluorescence behavior at the nanoscale and unravel the role of individual interactions that drive the observed enhancements.

### Single-Particle Investigation of CNC/PPE-CO_2_ Interactions

Single-particle spectroscopy measurements were performed using a stage-scanning inverted fluorescence microscope equipped with a pulsed 405 nm laser (Figure 3). A high numerical aperture objective (1.49 NA; 60X) collected the emission from single CNC/PPE-CO_2_ particles and directed the photons to an avalanche photodiode detector (APD), and they were processed using a time-correlated single-photon counter. The intensity versus time trajectories obtained from the single particles enabled the extraction of the fluorescence lifetime and probing of the spectral shift over time for the prepared fluorescent hybrid nanoparticles.

**Figure 3:**
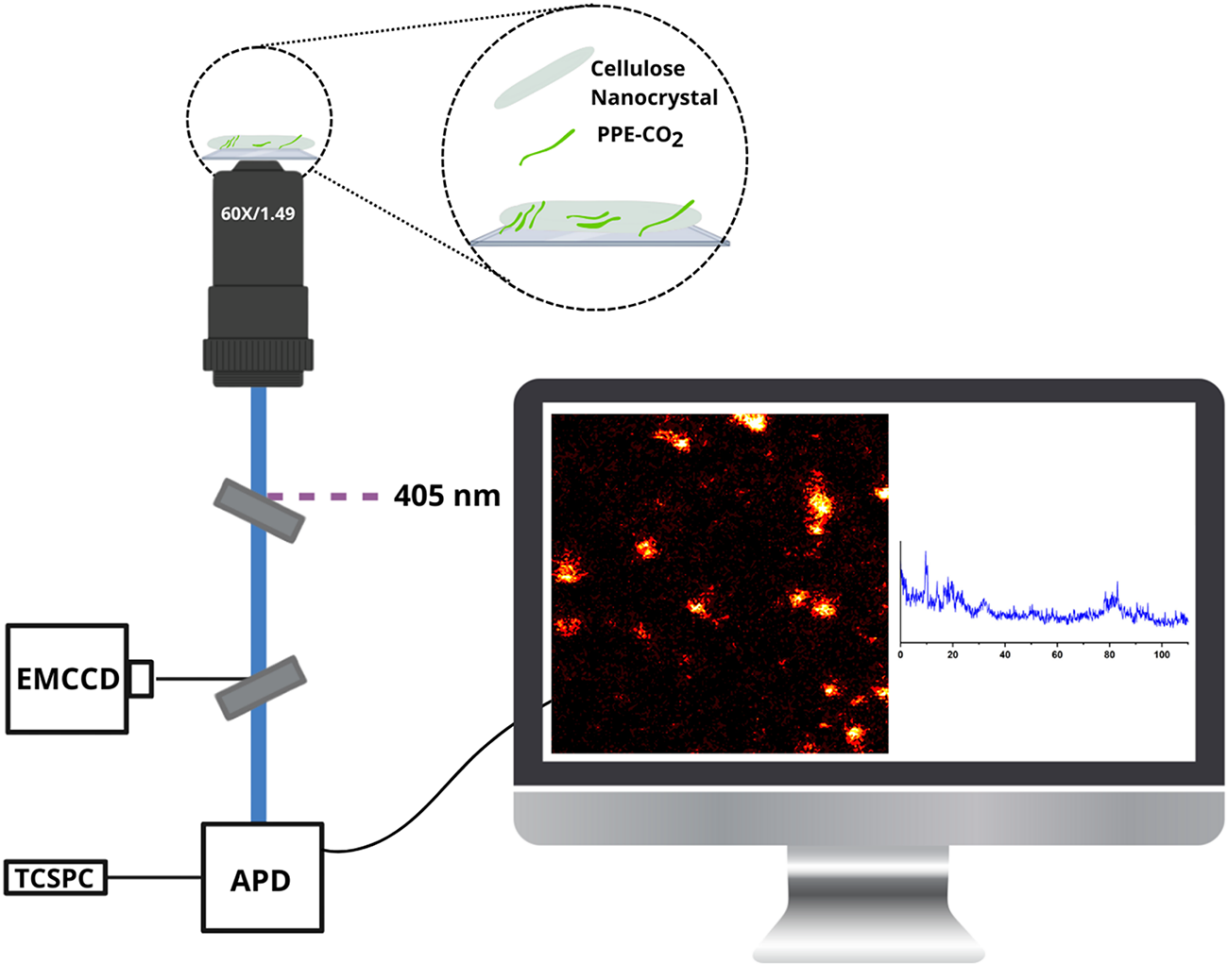
A schematic illustration of the fluorescent microscope equipped with stage scanning. Emission is collected by an objective and directed toward either an APD or diffracted by a monochromator into an EMCCD camera. A representative 10 × 10 μm^2^ single-particle fluorescence scanning confocal image was obtained upon 405 nm excitation of the CNC/PPE-CO_2_ assembled onto cellulose nanocrystals, and a representative intensity over time trajectory.

Initially, intensity-time trajectories for CNC/PPE-CO_2_-108 particles were acquired (Figure 4). These measurements provide valuable insights into the photophysical properties of conjugated polymers. While exciton migration and related processes occur on ultrafast time scales beyond our resolution, their structural consequences, such as blinking and cumulative photobleaching, manifest at longer observable time scales, offering specific information on exciton migration, trapping, and annihilation. Additionally, they can reveal conformation changes and solvation states in conjugated polymers, as the trajectories can be sensitive to changes in the local environment.^53^ The collected intensity-time traces were visually inspected and categorized into three different groups; Category 1 is characterized by a blinking behavior where the intensity jumped between different levels and remained relatively constant when dwelling at certain levels. Category 2 represents the trajectories where the intensity decreased steadily over time. Category 3 is designated for the traces whose intensity did not decrease nor showed blinking over the measurement time window. For CNCs/PPE-CO_2_-108, out of the 67 recorded trajectories, 60% exhibited blinking behavior, 27% demonstrated exponential decay, and 13% maintained a stable intensity over time. These single-particle observations reveal the inherent heterogeneity of conjugated polyelectrolytes, a well-known phenomenon when these systems are studied at the single-molecule level.^54^ In contrast to the ensemble measurements (Figure 2A), we did not observe an increase in the fluorescence intensity over time, even when excited at different laser powers (data not shown). This could be attributed to the fact that we are probing the emission from surface-bound hybrid nanoparticles, which limits the scission of conjugated polyelectrolytes and lacks species that emit from excimer-like states. While we cannot fully exclude contributions from photoinduced structural rearrangements, such conformational dynamics are less likely to occur under our experimental conditions.

**Figure 4:**
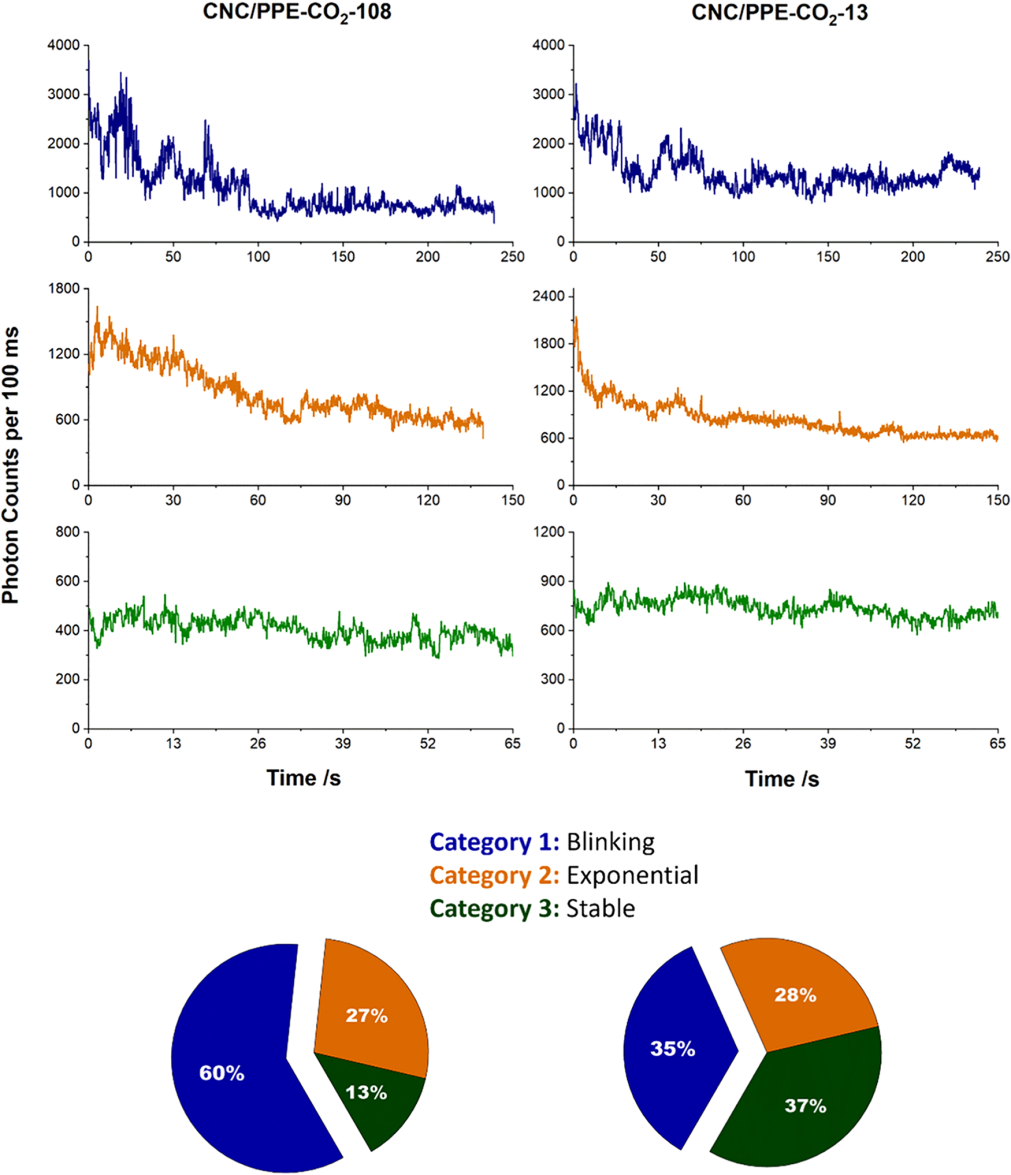
Representative single-particle intensity–time trajectories, binned at 100 ms, are shown for CNC/PPE-CO_2_-108 and CNC/PPE-CO_2_-13. The traces are grouped into three categories: (Category 1) blinking (blue), (Category 2) exponential decay, and (Category 3) constant intensity over time. The accompanying pie charts illustrate the distribution of these categories for each polymer length.

Previous single-molecule/particle intensity-time trajectories on both conjugated polymer and polyelectrolytes reported similar observations; blinking suggests an efficient exciton migration between the different chromophores in well-ordered conjugated polymer chains.^55–57^ In these systems, planarized chains promote strong interchain interactions, leading to extended conjugation lengths.^58^ In contrast, randomly arranged polymers exhibit continuous exponential photobleaching due to inefficient energy transfer between the different chromophores, resulting in random photobleaching of individual units.^56^

One of the earliest single-molecule studies on conjugated polymers, by Rothberg et al., demonstrated that MEH-PPV prepared from a poor solvent like toluene formed collapsed but ordered chains.^59^ The recorded intensity over time traces showed blinking. Conversely, when cast from a good solvent such as chloroform, the polymers adopted a more expanded colloidal structure, exhibiting exponential decay. Similar rapid intensity fluctuations have been observed in single P3HT chains, attributed to localized charges (deep exciton traps). These charges, with low mobility, remain confined in specific polymer regions, quenching emission locally and reducing the number of emitters.^60^ These earlier observations were limited to the studies of drop-casted conjugated polymers. However, even when P3HT polymers were anchored to the glass surface and studied in DMSO (poor solvent),^61^ fast blinking in the fluorescent intensity was also observed and attributed to a large number of chromophores undergoing reversible transit to dark states because of the restricted conformation freedom of the single chains in the poor solvent. As such, these chains had enough disorder to have localized charge states but not enough to undergo exponential photobleaching. When conjugated polyelectrolyte MPS-PPV was encapsulated as a freely diffusing yet coiled polymer in negatively charged liposomes and studied at the single molecule level, discrete fluorescence intensity jumps, and individual on–off transition steps were reported.^55^ This quantized behavior revealed an efficient interchain exciton transport to localized fluorescence quenching sites along the PPV backbone (oxidation, kinks, etc.). Since the majority of the traces revealed blinking behavior, it indicates that PPE-CO_2_ was assembled onto the CNC as ordered assemblies. A different perspective was reported by Vacha et al. where they showed that blinking could be the result of the interaction of the polymer chain quenchers localized on the surface of the substrate. By comparing the intensity-time trajectory of MEH-PPV of freely diffusing polymer in solution and adsorbed on a polystyrene surface, they reported complete suppression of blinking in solution, while 94% of the traces of the adsorbed molecules showed either stochastic blinking or blinking with an exponential decay pattern.^62^ If the cellulose nanocrystals’ surface has a direct impact, similar to what Vacha et al. reported, decreasing the conjugated polymer length should theoretically affect the intensity-time traces. The fluorescence emission spectrum of CNC/PPE-CO_2_-13 in an aqueous suspension also shows suppressed excimer emission and enhanced fluorescence (Figure S6). CNCs/PPE-CO_2_-13 hybrid particles were then prepared and imaged at the single-particle level. Among the 57 analyzed trajectories, 35% exhibited blinking, which is much lower than that reported for 108 (60%). Interestingly, 37% displayed a stable intensity change much higher than that of the CNC/PPE-CO_2_-108. While we do not fully understand the reason behind this improvement, it will be the subject of future investigations since highly stable and nonblinking behavior could be instrumental in imaging applications. Lastly, 28% demonstrated exponential decay.

As a summary, we believe the observed blinking in the CNC/PPE-CO_2_ could be the result of ordered and densely packed conjugated polyelectrolytes onto the surface of the nanocrystals. The single-step photobleaching reveals an efficient energy transfer between neighboring chromophores, which is then quenched by defective sites. The larger PPE-CO_2_ allows efficient interchain exciton transfer over larger polymer packing volumes and more interaction with the nanoparticle surface.

To confirm this nanostructural polymer conformation onto the cellulose nanocrystals, we extracted the fluorescence lifetime from the single particle intensity measurements. We observed a striking difference in the fluorescence lifetimes between the free polymers and those assembled onto the cellulose nanocrystals’ surface; the CNC/PPE-CO_2_ complexes prepared in a buffer solution exhibited a lifetime similar to those measured from free CPEs in deionized water (Table 1). In deionized water, the PPE-CO_2_ emits mostly from single polymers with predominantly short lifetimes with 0.317 ns (Relative Amplitude­(RA) = 0.6) and 0.390 ns (RA = 1) for PPE-CO_2_-13 and 108, respectively, due to the formation of nonaggregated species stabilized by the lack of a counterion. However, upon the incremental addition of Na^+^ ions, the electrostatic repulsion between the individual polymer side groups is reduced by shielding the backbone charges. It then favors aggregate formation and emission from excimer-like emission with longer lifetimes.^63,64^ PPE-CO_2_ prepared in 10 mM HEPES and 150 mM NaCl exhibited a predominantly long lifetime of 3.77 ns (RA = 0.68) and 2.40 ns (RA = 0.64) for 13 and 108 average repeating units, respectively. When cellulose nanocrystal particles were added to the PPE-CO_2_ prepared in a buffer solution, we observed a shift toward a shorter lifetime similar to that measured in DI. The CNC/PPE-CO_2_-13 complex revealed a biexponential decay, characterized by a dominant short lifetime of 0.470 ns (RA = 0.64), alongside a longer lifetime of 1.94 ns. On the other hand, the CNC/PPE-CO_2_-108 complex exhibited a monoexponential decay with a lifetime of 0.450 ns. These results confirm that the PPE-CO_2_ chains are not aggregated onto the cellulose nanocrystal’s surface in a conformation that would emit from excimer-like species.

**Table 1:** Summary of the Fluorescence Lifetime Measurements of PPE-CO_2_-13 and 108, in DI, in Buffer (10 mM HEPES and 150 mM NaCl), and When Assembled Onto Cellulose Nanocrystal Surfaces. RA_1_ and RA_2_ Are the Calculated Relative Amplitudes

	RA_1_	τ_1_ (ns)	RA_2_	τ_2_ (ns)
PPE-CO_2_-13 (DI)	**0.60**	**0.317**	**0.40**	**2.73**
PPE-CO_2_-13 (Buffer)	0.32	0.518	0.68	3.77
PPE-CO_2_-13 (Buffer)/CNC	**0.64**	**0.470**	**0.36**	**1.94**
PPE-CO_2_-108 (DI)	*1*	*0.390*		
PPE-CO_2_-108 (Buffer)	0.68	2.40	0.32	3.12
PPE-CO_2_-108 (Buffer)/CNC	*1*	*0.450*		

To better understand the unique photophysical changes of the CNC/CPEs complexes when irradiated over time, emission spectra from single particles were acquired over time with an integration of 10 s (Figures 5 and S7). Unlike the ensemble spectra in Figure 1B, which represent the average emissions from the aqueous suspensions, the single-particle spectra in Figure 5 are recorded from immobilized nanoparticles in the dried state, thus revealing heterogeneity and spectral features, otherwise averaged out. These emissions revealed two dominant species: a red species with a maximum emission at around 500 nm, indicating a more extended or ordered conjugated polymer, and a blue species centered at 450 nm overlapping with the typical emission of well-dissolved single PPE-CO_2_, both with well-structured emission spectra and far from the typical structureless emission of excimer-like species at 520 nm.

**Figure 5:**
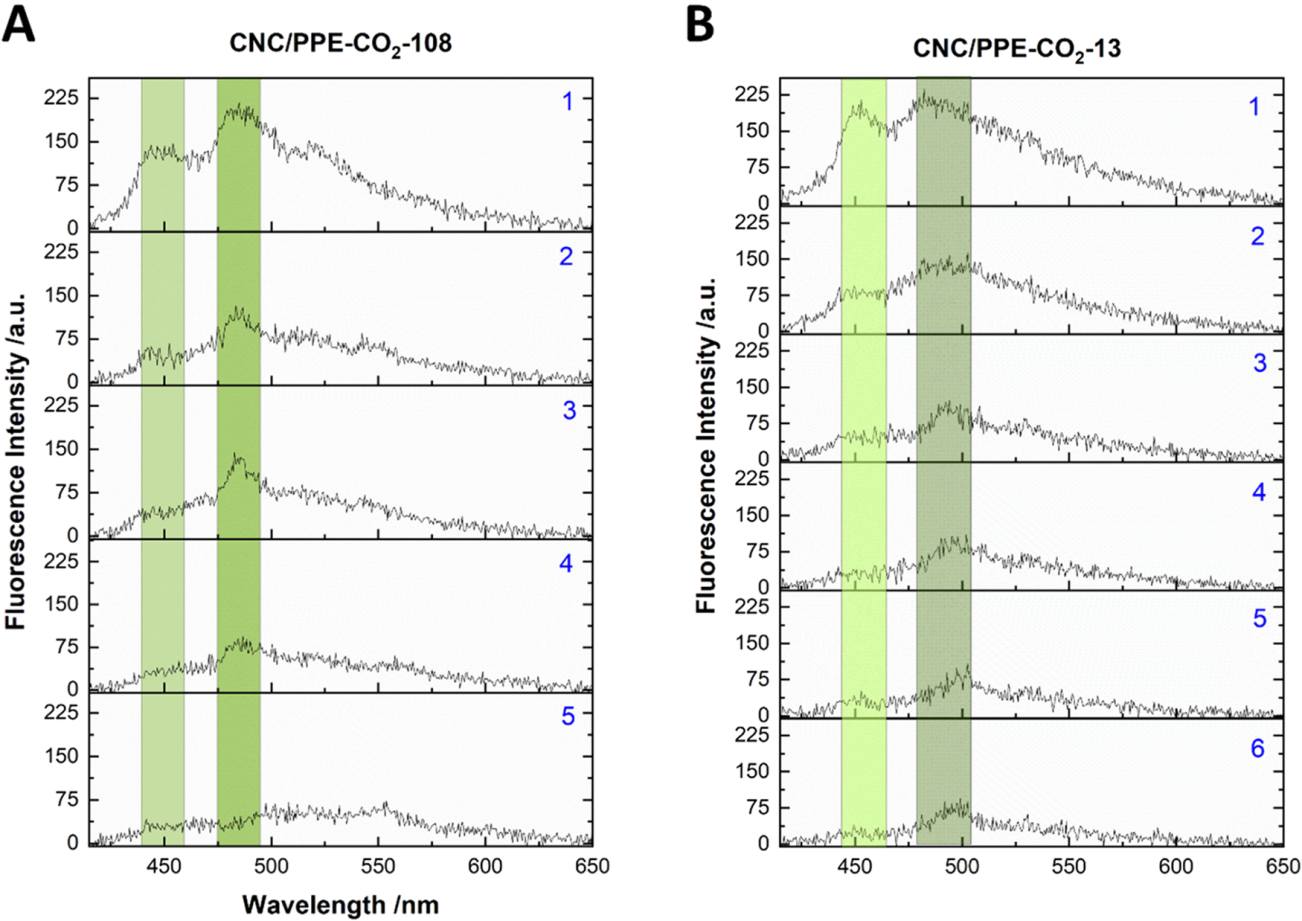
Representative fluorescence emission from a single particle of (A) CNC/PPE-CO_2_-108 and (B) CNC/PPE-CO_2_-13. Each spectrum was integrated for 10 s, capturing the emission over time as the particle undergoes photobleaching.

Interestingly, the blue peak at 450 nm photobleached first during the intensity photodegradation. This behavior aligns with the fact that individual short polymers would photobleach abruptly, while ordered assemblies would show longer photodegradation due to the sequential photobleaching of the multiple chromophores. Similar results were observed by Koufman et al., who studied the change in the fluorescence emission of single MEH-PPV before and after solvent vapor annealing (SVA) when trapped in a PMMA matrix.^65^ Initially, the majority of the collected emissions had a maximum emission peak at around 545 nm. While they did not report the change in the intensity over time, however, they observed that after SVA, the single MEH-PPV polymer chains exhibited a red-shifted emission with a 0–0 peak at around 575 nm. This shift was attributed to the formation of ordered aggregates where interchain interactions enable the fast exciton migration toward a lower energy state, resulting in a red-shifted emission.

## Conclusion

Using cellulose nanocrystals, we prepared highly fluorescent and photostable hybrid nanoparticles. In a buffer solution, PPE-CO_2_ is aggregated in conformations that promote emission from excimer-like species. This conformation state has poor photophysical properties, such as low emission and fast photodegradation. The addition of cellulose nanocrystals to the PPE-CO_2_ polymer leads to a substantial enhancement in their photophysical properties, with reported highly emissive and photostable particles. These enhancements are believed to be the result of the change in the PPE-CO_2_ aggregation state into well-ordered packed conformations. This work presents a new insight into the unique photophysical properties of CPEs and how their photophysical properties could be tuned by cellulose nanocrystals. It also showcases the potential cellulose nanocrystals have in enhancing the properties of fluorescent materials. While our work demonstrates the enhanced photophysical properties of CPEs when assembled onto CNC nanoparticles, future studies will provide deeper insight into the underlying structural dynamics responsible for some of these observed enhancements. By combining spectroscopic measurements with molecular simulations, we will unravel the precise bonding mechanism and elucidate the structural organization of the CPEs on the CNC surface to establish a generalized design principle.

## Materials and Methods

### Materials

Poly­(phenylene ethynylene) carboxylate (PPE-CO_2_) with average monomer repeating units of 13 and 108 was synthesized using the Sonagashira coupling method, following previously reported protocols.^66,67^ 2-[4-(2-Hydroxyethyl)­piperazin-1-yl]­ethanesulfonate sodium salt (HEPES) and sodium chloride (NaCl) were obtained from Fisher Scientific. Deionized water with a resistivity of 18.2 MΩ·cm was utilized in all experiments. Cellulose was purchased from CelluForce Inc. (NCV100-NASD90 CelluForce NCC) as a sulfate salt with a reported size of less than 150 nm.

### Steady-State Measurements

All steady-state fluorescence measurements were conducted using a Horiba Fluorolog-3 fluorometer with a T3 Quantum Northwest temperature controller. The excitation wavelength was set to either 405 or 420 nm, and fluorescence spectra were recorded from 420 to 650 nm or 435 to 650 nm, respectively. Absorbance measurements were taken from 250 to 600 nm using a Jasco V-570 UV/vis/NIR spectrophotometer at room temperature unless otherwise specified. The solutions contained 3 μg/mL of either PPE-CO_2_-108 or PPE-CO_2_-13 prepared in a buffer solution of 10 mM HEPES with 150 mM NaCl.

### Single-Particle Measurements

A custom-built confocal fluorescence microscopy system (Olympus IX71) with a high-numerical-aperture APON 60× objective (NA = 1.49) was used in all single particle experiments. The cellulose nanocrystals/PPE-CO_2_ samples, prepared under varying experimental conditions, were excited at 405 nm using a fiber-coupled diode laser (PicoQuant GmbH, LDH-C-405) operating in quasi-continuous wave mode with pulsed excitation at an 80 MHz repetition rate. To ensure purity, the excitation beam was filtered through an HC Laser Clean-up 405/10 filter (AHF analysentechnik AG) and circularly polarized via a quarter-wave (λ/4) plate. A single-photon avalanche photodiode (Micro Photon Devices S.r.l., PDM series) detected the fluorescence signal, which was processed using a time-correlated single-photon counting (TCSPC) module (PicoQuant GmbH, HydraHarp 400) to extract fluorescence lifetime data.

Fluorescence emission was collected by focusing the laser beam on the cellulose nanocrystals/PPE-CO_2_ particles. The emitted light was focused onto a diffraction grating monochromator and was then recorded by an EMCCD camera.

## Supplementary Material



## Notes

Additional ensemble and single particle fluorescence data (PDF)
