# Supplementary material for: Surface-Induced Photophysical Enhancement of Conjugated Polyelectrolytes on Cellulose Nanocrystals: A Single-Particle Study
Source: Langmuir. 2025 Oct 10;41(41):28062–71. doi: 10.1021/acs.langmuir.5c03918 (PMC12548090; doi:10.1021/acs.langmuir.5c03918)
Supplement: Supplementary file 1 [file la5c03918_si_001.pdf]

Supporting Information

Surface-Induced Photophysical Enhancement of Conjugated Polyelectrolytes on Cellulose Nanocrystals: A Single-Particle Study

*Nour Merhi, Pierre Karam**

Department of Chemistry, American University of Beirut, P.O. Box 11-0236, Beirut, Lebanon

*To whom correspondence should be addressed:

Phone: 961-1-350000 Ext 3989 **Fax:** 961-1-350000 Ext 3970 **Email:** pk03@aub.edu.lb

Table of Figures:

Figure S1: A) Fluorescence emission spectra and B) Time-resolved fluorescence trajectory of conjugated polyelectrolyte PPE-CO_2-108 with increasing concentrations of nanocellulose in HEPES with NaCl. The trajectory was recorded at an emission wavelength 450 nm. 2
Figure S2: A) Absorption spectra of PPE-CO_2-108 in HEPES with NaCl after the addition of 4.5 mg/mL cellulose nanocrystals (blue), and in grey PPE-CO_2 in methanol. (B) Fluorescence emission spectra of PPE-CO_2-108 after the addition of 4.5 mg/mL cellulose nanocrystals (blue), and in grey PPE-CO_2 in methanol. 3
Figure S3: Time-dependent absorption and emission kinetics of CNC/PPE-CO_2 hybrids under continuous irradiation. 3
Figure S4: Emission spectra of PPE-CO_2-108 before and after photobleaching.........................4
Figure S5: Normalized emission spectra of CNC/PPE-CO_2-108 before and after continuous irradiation. 5
Figure S6: Fluorescence emission spectra of PPE-CO_2-13 before (green) and after (orange) the addition of 4.5 mg/mL cellulose nanocrystals. 6
Figure S7: Representative fluorescence emission from a single particle of CNC/PPE-CO_2-108 (S7A and S7B) and CNC/PPE-CO_2-13 (S7C and S7D). Each spectrum was integrated for 10 seconds, capturing the emission over time as the particle undergoes photobleaching. 7

1- Investigating the Interaction between Cellulose Nanocrystals (CNC) and the Conjugated Polyelectrolyte PPE-CO_2-18 using Emission Spectroscopy and Time-resolved Fluorescence Trajectory

To understand the interaction between nanocellulose and PPE-CO_2-108, we measured the change in fluorescence intensity with incremental additions of nanocellulose. In the steady-state fluorescence spectra, increasing nanocellulose concentrations led to an enhancement in fluorescence intensity, particularly at shorter wavelengths, indicating an interaction between the two components (Figure S1A). Additionally, time-dependent fluorescence measurements showed a gradual increase in fluorescence intensity with higher nanocellulose concentrations, suggesting an interaction that enhances fluorescence over time (Figure S1B). The increase in fluorescence intensity with nanocellulose provides evidence of an interaction.

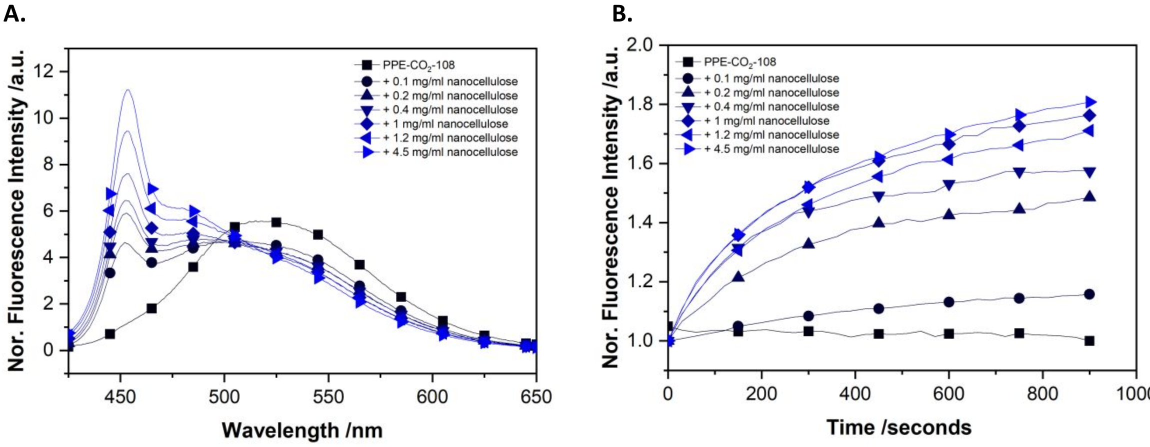

Figure S1: A) Fluorescence emission spectra and B) Time-resolved fluorescence trajectory of conjugated polyelectrolyte PPE-CO_2-108 with increasing concentrations of nanocellulose in HEPES with NaCl. The trajectory was recorded at an emission wavelength 450 nm.

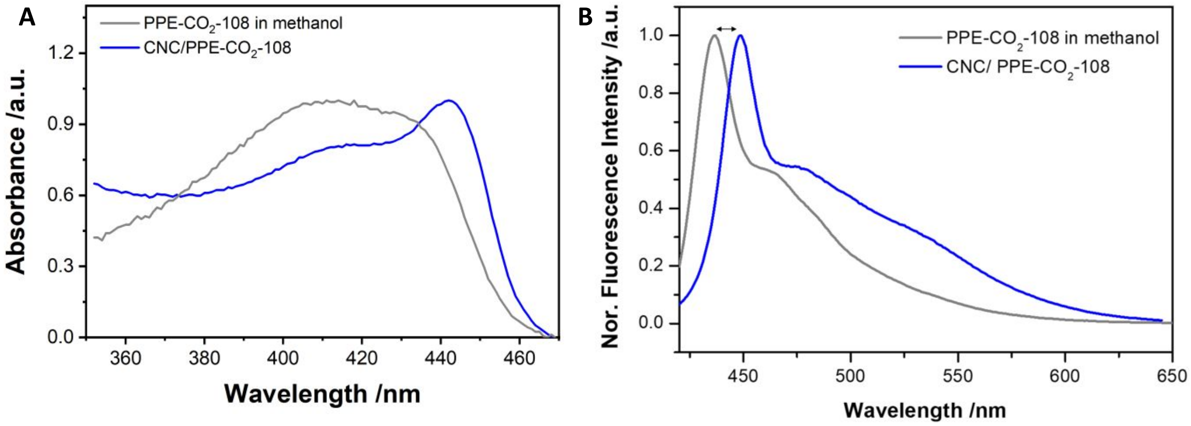

Figure S2: A) Absorption spectra of PPE-CO_2-108 in HEPES with NaCl after the addition of 4.5 mg/mL cellulose nanocrystals (blue), and in grey PPE-CO_2 in methanol. (B) Fluorescence emission spectra of PPE-CO_2-108 after the addition of 4.5 mg/mL cellulose nanocrystals (blue), and in grey PPE-CO_2 in methanol.

Additional spectroscopic measurements were performed to study the interaction between the CPE and the CNC nanoparticles (Figure S3). The absorption peak at 442 nm increased upon CNC addition and remained stable over time. Similarly, the non-excimeric emission band at 450 nm also increased upon the addition of CNC and continued to increase over time, which supports our interpretation that irradiation selectively deactivates low-energy quenching sites.

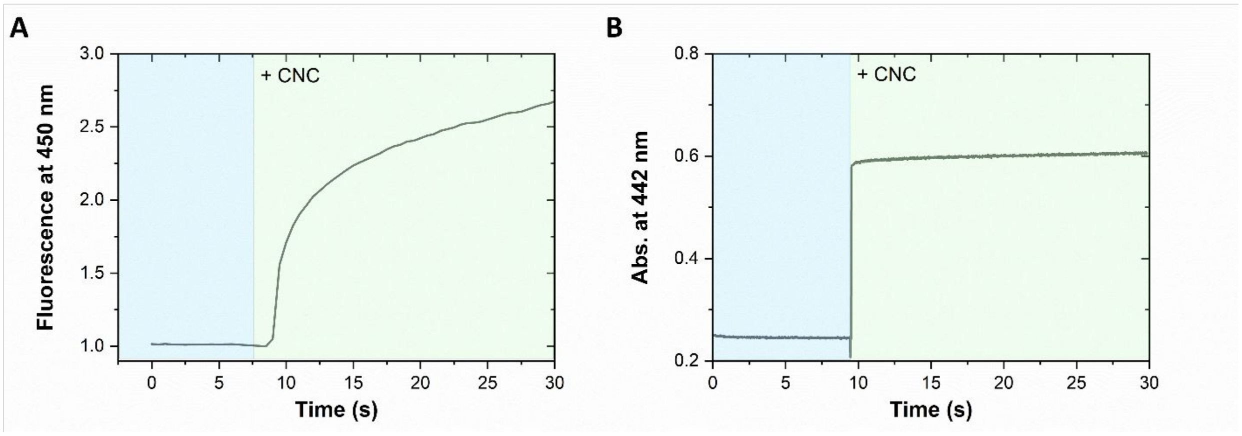

Figure S3: Time-dependent (A) emission and (B) absorbance kinetics of CNC/PPE-CO_2 hybrids under continuous irradiation.

2- Fluorescence Emission of CNC/PPE-CO_2-108 Upon Subjecting the Assembly to Continuous Irradiation for 1 hour

Upon continuous irradiation of PPE-CO_2-108 and CNC/PPE-CO_2-108 assemblies for an hour, a change in the fluorescence behavior was observed (Figures S4 and S5). The reduction of red emission may be indicative of the disruption of excimer-like species. These findings suggest that the destruction of low-energy quenching sites upon prolonged exposure results in a more emissive system with reduced contributions from lower-energy, excimer-related states.

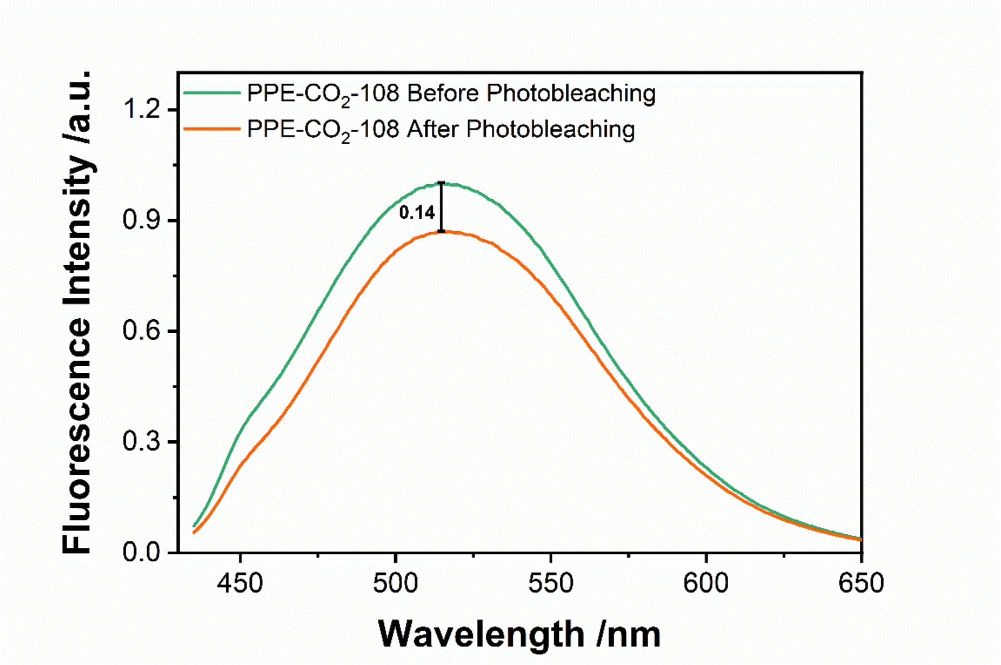

Figure S4: Emission spectra of PPE-CO_2-108 before and after photobleaching.

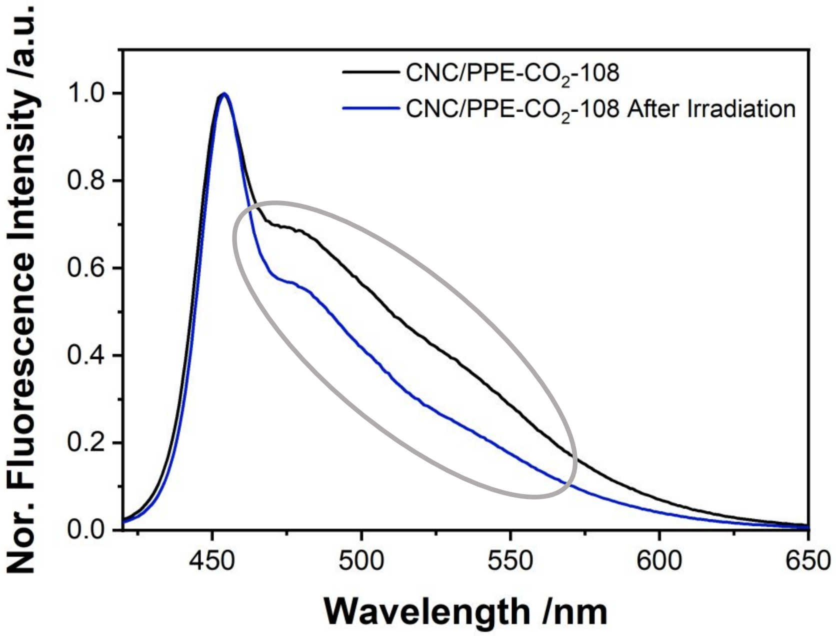

Figure S5: Normalized emission spectra of CNC/PPE-CO_2-108 before and after continuous irradiation.

3- Time-Resolved Single-Particle Emission Analysis of CNC/PPE-CO_2 Complexes under Continuous Irradiation

To gain deeper insights into the distinct photophysical behavior of CNC/CPE complexes under continuous irradiation, emission spectra from individual particles were collected over time using a 10-second integration period. The spectral evolution revealed the presence of two main emissive populations: a red-emitting species peaking near 500 nm, suggestive of a more ordered or extended conjugated polymer structure, and a blue-emitting species centered around 450 nm, corresponding to the characteristic emission of well-dissolved single PPE-CO_2 chains. Notably, the blue emission exhibited sharp, structured features, distinguishing it from the broader, structureless excimer-like emission typically observed near 520 nm. (Figures S7)

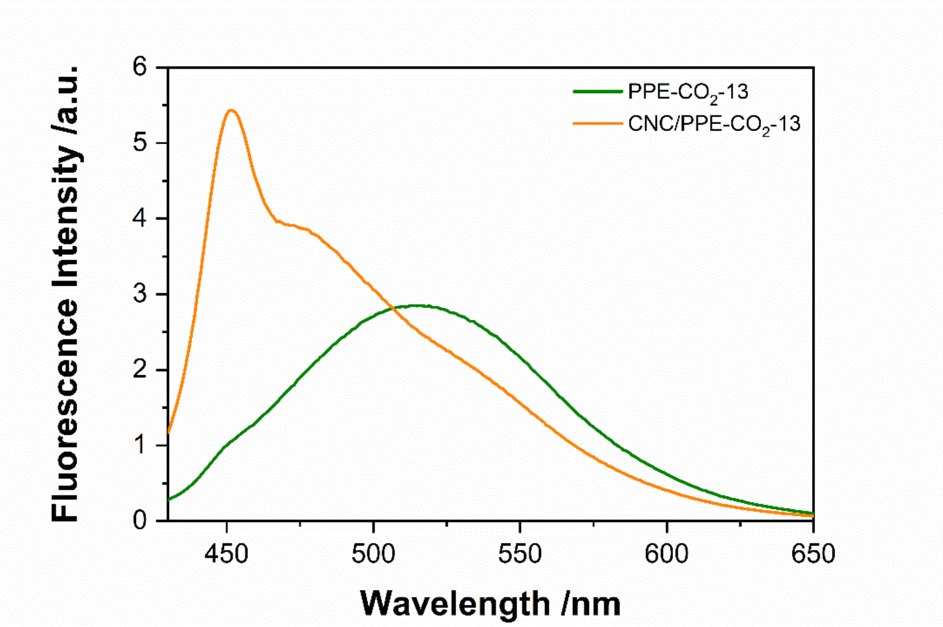

Figure S6: Fluorescence emission spectra of PPE-CO_2-13 before (green) and after (orange) the addition of 4.5 mg/mL cellulose nanocrystals.

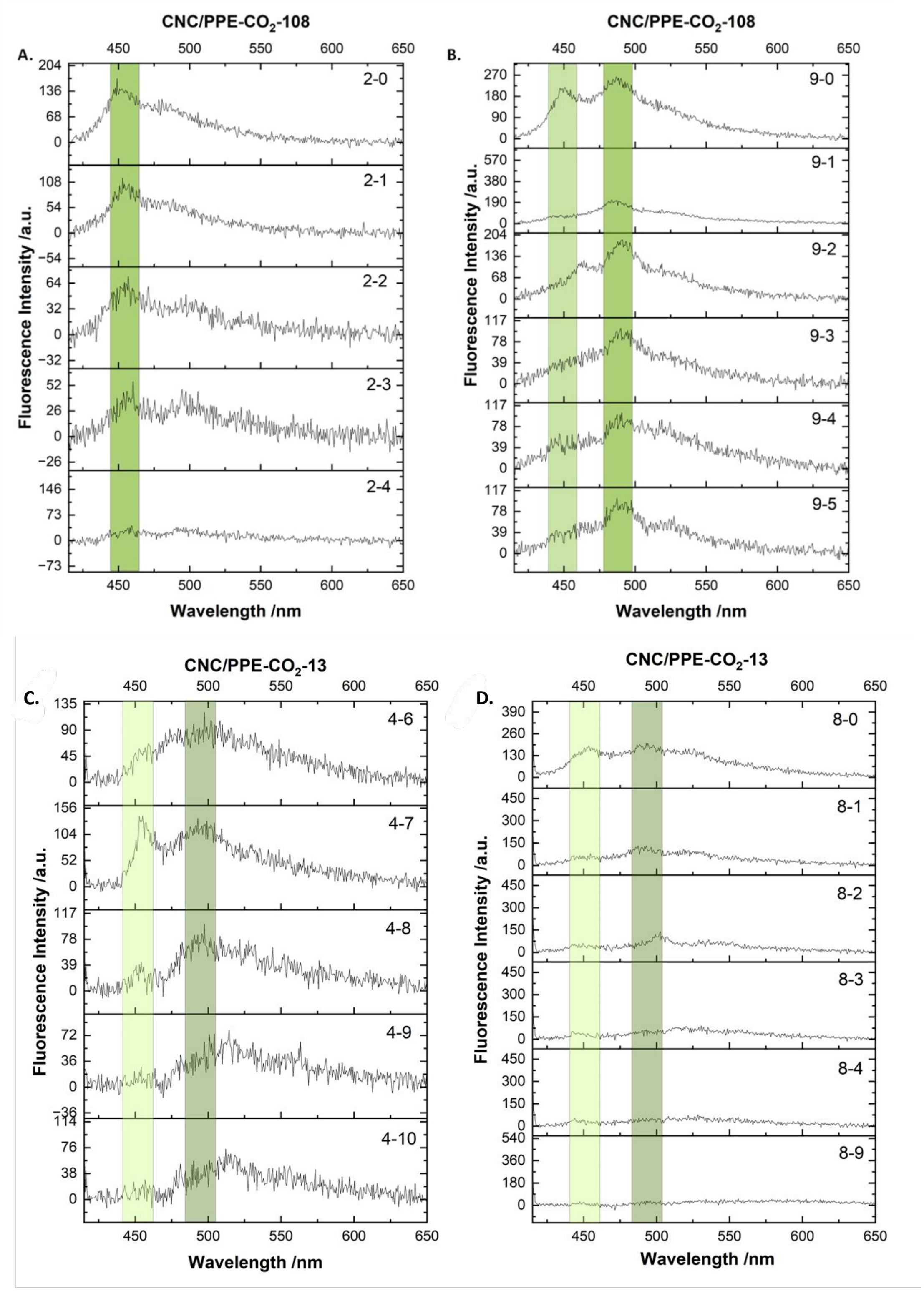

Figure S7: Representative fluorescence emission from a single particle of CNC/PPE-CO_2-108 (A and B) and CNC/PPE-CO_2-13 (C and D). Each spectrum was integrated for 10 seconds, capturing the emission over time as the particle undergoes photobleaching.
